# Follistatin attenuates radiation-induced fibrosis in a murine model

**DOI:** 10.1371/journal.pone.0173788

**Published:** 2017-03-16

**Authors:** Helen B. Forrester, David M. de Kretser, Trevor Leong, Jim Hagekyriakou, Carl N. Sprung

**Affiliations:** 1 Centre for Innate Immunity and Infectious Diseases, Hudson Institute of Medical Research, Clayton, Victoria, Australia; 2 Department of Molecular and Translational Science, Monash University, Clayton, Victoria, Australia; 3 Centre for Reproductive Health, Hudson Institute of Medical Research, Clayton, Victoria, Australia; 4 Department of Anatomy and Developmental Biology, Monash University, Clayton, Victoria, Australia; 5 Division of Radiation Oncology and Cancer Imaging, Peter MacCallum Cancer Centre, East Melbourne, Victoria, Australia; Northwestern University Feinberg School of Medicine, UNITED STATES

## Abstract

**Purpose:**

Fibrosis can be a disabling, severe side effect of radiotherapy that can occur in patients, and for which there is currently no effective treatment. The activins, proteins which are members of the TGFβ superfamily, have a major role in stimulating the inflammatory response and subsequent fibrosis. Follistatin is an endogenous protein that binds the activins virtually irreversibly and inhibits their actions. These studies test if follistatin can attenuate the fibrotic response using a murine model of radiation-induced fibrosis.

**Experimental design:**

C57BL/6 mice were subcutaneously injected with follistatin 24 hours prior to irradiation. Mice were irradiated in a 10 x 10 mm square area of the right hind leg with 35 Gy and were given follistatin 24 hours before radiation and three times a week for six months following. Leg extension was measured, and tissue was collected for histological and molecular analysis to evaluate the progression of the radiation-induced fibrosis.

**Results:**

Leg extension was improved in follistatin treated mice compared to vehicle treated mice at six months after irradiation. Also, epidermal thickness and cell nucleus area of keratinocytes were decreased by the follistatin treatment compared to the cells in irradiated skin of control mice. Finally, the gene expression of transforming growth factor β1 (Tgfb1), and smooth muscle actin (Acta2) were decreased in the irradiated skin and Acta2 and inhibin βA subunit (Inhba) were decreased in the irradiated muscle of the follistatin treated mice.

**Conclusions:**

Follistatin attenuated the radiation-induced fibrotic response in irradiated mice. These studies provide the data to support further investigation of the use of follistatin to reduce radiation-induced fibrosis in patients undergoing radiotherapy for cancer.

## Introduction

Normal tissue reactions in response to radiotherapy can be broadly classified into two distinct subtypes; ‘acute’ and ‘chronic (late)’. Acute reactions occur during or soon after radiotherapy and late effects occur months to years after irradiation. Acute reactions can be managed during radiotherapy as treatment can be halted to prevent unacceptable adverse reactions. However, the late reactions that develop in patients only emerge long after radiotherapy has been completed. Since these adverse effects are not predictable in patients before treatment, the radiation doses used in clinical practice are conservative and thus some patients who could potentially tolerate higher radiation doses do not receive the maximum benefit from radiotherapy. Some of these late radiation-induced fibrotic reactions are debilitating and can result in death if they occur in critical organs such as the lung or liver.

Radiation-induced fibrosis results from the inflammatory response induced by radiation, which involves the activation of myofibroblasts to produce excessive deposits of collagen and other extracellular matrix components [1]. The magnitude of all fibrosis-induced mortality is such that it has been proposed to be the cause in 45% of all natural deaths in the USA [2]. The major adverse reactions following thoracic radiotherapy, for example, are pneumonitis and pulmonary fibrosis [3] where death is a possible outcome. The risk of developing these adverse reactions is related to radiation dose, irradiated volume and fractionation schedule [4], but also has a genetic component [5].

Oxidation and cellular damage due to radiation causes an inflammation response [6]. The inflammatory response to radiation is complex and involves local cellular and molecular events including the release of cytokines and growth factors. Immune cells such as macrophages are recruited, and myofibroblasts are activated. Part of the repair process that follows the initial inflammation involves laying down extracellular matrix scaffold which supports regrowth of tissue. Following tissue regrowth, the repair process is resolved and the extracellular matrix is remodelled. Fibrosis occurs when inflammation continues and the repair process is not resolved. A self-sustaining cycle of inflammation and chronic oxidative stress ensues that can lead to fibrosis [6] for which genetics can play an important role in sensitivity in the case of radiotherapy [7]. These genetic factors contribute to the severity of the radiation-induced inflammatory / fibrotic response, and can partially explain why patients who undergo the same radiotherapy regime show differences in adverse tissue responses [7].

A major goal in the field of radiation oncology research is to find a way to diminish the severity of fibrosis caused by radiotherapy. Currently there is no effective treatment for fibrosis in clinical practice, and the discovery of an effective treatment is a major objective in the field of radiation oncology.

Transforming Growth Factor β (TGFβ) is recognised as a key player in the development of fibrosis [8] but recent data has implicated the activins A and B in the fibrotic response [9,10]. This data arose from a study which showed that follistatin could attenuate the actions of TGFβ, despite the fact that follistatin cannot bind TGFβ [11]. Previous studies have shown that TGFβ induces fibroblasts to produce activin A [12]. Also activin A stimulates extracellular matrix proteins such as collagens and smooth muscle actin [13].

Activin A is a disulphide-linked dimer of the inhibin βA subunits which form activin A (β_A_β_A_) [14]. Activin A stimulates inflammation and fibrosis in many different models such as inflammatory bowel disease, rheumatoid arthritis, following burns injuries and in wound healing [10]. A key regulator of activin is follistatin, a glycoprotein that binds the activins virtually irreversibly, targeting the complex to a lysosomal degradation pathway [13]. Follistatin is expressed as two major protein isoforms, FST-288 and FST-315 through alternative splicing of a single gene. Both isoforms circulate but FST-315 is found at higher levels than FST-288. FST-288 spontaneously binds to heparan sulphate proteoglycans on cell surfaces *via* a positively charged heparin binding site [15] and FST-315 can bind to cell surfaces after binding activin due to a change in its tertiary structure [12,16]. In mice, blocking the actions of activin A released following lipopolysaccharide exposure using follistatin alters the induced cytokine cascade attenuating the rise of TNFα and altering the levels of other inflammatory cytokines (e.g., IL1β and IL-6) [9]. This study established the importance of activin A as a key regulator of the inflammatory response. Like TGFβ, activin A acts via serine / threonine kinase receptors involving dimerisation of a type II receptor and an activin receptor-like kinase type I receptor [17] and activates phosphorylation of the transcription factors SMAD2 / 3. The functional value of the activins is apparent from the highly conserved amino acid sequence of the β subunits between humans and rodents (100% for β_A_; 97% for β_B_) [18,19].

Follistatin is also highly conserved with 97% amino acid sequence similarity across a range of mammals including humans and rodents [18,20]. In addition to binding the activins, follistatin also binds to other TGFβ superfamily proteins including GDF8, GDF9, and a number of bone morphogenic proteins (BMPs 2,5,7 & 8), however, binding with these proteins has affinities at least 10-fold lower than that for activin [19]. Follistatin does not bind to TGFβ1 or β2 but has been reported to bind to TGFβ3 [21]. Follistatin can block the fibrogenic actions of TGFβ despite its inability to bind TGFβ1 suggesting that TGFβ1 is exerting its fibrogenic actions through its stimulation of activin A [10]. Other factors considered to be important in fibrosis are also induced by activins; these include: TNFα, CTGF, COL1A1, TIMP-1, PAI-1 and endothelin [10], some of which are also induced by TGFβ [22]. At the cellular level, activin A also induces the differentiation of monocytes into activated macrophages which can promote fibrosis [10]. As follistatin binds the activins, it attenuates the capacity of activin A to stimulate fibrosis as demonstrated in CCl_4_-induced hepatic fibrosis in mice resulting from activin A secretion by hepato-stellate cells [23]. Furthermore, follistatin diminished bleomycin-induced pulmonary fibrosis in mice by attenuating the proinflammatory and profibrotic actions of activin A [24], and low expression levels have been associated with sensitivity to radiation-induced fibrosis [25,26] and Dupuytren’s disease, which is a condition associated with fibrosis of the palmar fascia [27]. Follistatin gene expression is also induced by radiation [25].

Therefore, the activin / follistatin pathway offers an effective novel biological target for the prevention of radiation-induced fibrosis. This study establishes that follistatin can ameliorate the fibrotic response in an *in vivo* model of radiation-induced fibrosis.

## Methods

### Animal studies

To study the role of the activins and follistatin in radiation-induced fibrosis, two groups of eight week old C57BL/6 male mice (n = 9 per group) obtained from Monash Animal Services were used. The right hind legs were exposed to radiation with one group receiving only phosphate buffered saline (PBS) and the other receiving follistatin. For radiation exposure, the mice were anaesthetised with 10 mg / ml ketamine and 1 mg / ml xylazine (0.1 ml / 10 g intraperitoneal injection) and positioned (with tape to hold the right leg in place) in a custom jig to allow exposure to ionizing radiation in a 10 x 10 mm square area of the right hind leg with 35 Gy at a dose rate of 1 Gy / 5.1 sec generated by a conventional linear accelerator radiotherapy machine (Clinac, Varian) while the rest of the mouse (including the left leg) was protected by lead and plexiglass shielding.

To assess the effect of follistatin treatment, the mice in the experimental group were given 4 μg of human recombinant follistatin-288 in PBS subcutaneously in the abdomen 24 hours prior to irradiation, 2 days after the radiation treatment, and then three times per week over the course of six months. Mice were monitored at least three times per week for general health and adverse reactions to treatment. Mice were provided with unlimited regular pellet food and water during the course of the experiment and husbandry was provided by the Monash Animal Research Platform with a maximum of five mice per box. Control mice received injections of PBS subcutaneously. Human recombinant follistatin 288 was purified by chromatography from HEK293E cells stably expressing the EBNA protein (293EBNA cells), transfected with the human follistatin-expressing plasmid, pSV2HF288.

At the termination of the experiment (6 months after irradiation), the mice were anaesthetized with 10 mg / ml ketamine and 1 mg / ml xylazine (0.1ml / 10g). The amount of tissue contraction resulting from fibrosis was determined by measuring leg extension to determine if follistatin provided any beneficial effects on leg flexibility. Leg extension was measured by a single researcher using a customized jig with a platform and a fixed angle gauge to measure angle of extension from the vertical position in anaesthetized mice. Blood was then collected by cardiac puncture, allowed to clot at room temperature for at least 30 minutes and then centrifuged at 1000 g for 10 minutes to remove the clot. Serum levels of follistatin were measured by radioimmunoassay [28]. Skin and muscle tissue (gastrocnemius) was obtained from the site of irradiation and from the same region on the opposite non-irradiated leg for use as a non-irradiated control. The specific protocols used in this study were approved by the Monash University Animal Ethics committee (Approval Number MMCA-2011-44).

### RNA isolation

Tissues were snap frozen until RNA isolation. Tissue was resuspended in 0.5 ml of TRIsure (Invitrogen, Carlsbad, CA, USA) and homogenized with a Precelly homogenizer using 2.8 mm ceramic beads, sheared with 18 gauge needle and the aqueous phase collected after centrifugation. The aqueous layer was mixed with 120 μl chloroform, centrifuged and the aqueous phase collected as before. The RNA was precipitated with isopropanol, washed with 75% ethanol and resuspended in water. RNA was further purified on an RNeasy column (Qiagen, Venlo, Netherlands). The RNA extraction was continued by using the RNAeasy method as per manufacturer’s recommendation with the addition of a DNase1 digestion step. RNA concentration was determined by analysing on a nanodrop ND-1000 spectrophotometer (Thermo Scientific, Wilmington, Delaware, USA).

### Transcriptional validation

Primers were designed to candidate exons or genes using ‘primer 3’ or Primer-Blast on-line software (NCBI). cDNA was made from 500 ng RNA using Super Script III First-Strand Synthesis System for RT-PCR (Invitrogen, Carlsbad, USA) as per manufacturers recommendation and described previously [25]. Real-time PCR was performed using these primers under the following conditions: Sybr Green Master Mix (Applied Biosystems, United Kingdom) with 200 nM of each primer was mixed with 5 ng of cDNA. The cycling steps were as follows. 95°C: 10 min; (95°C: 15 sec; 60°C: 60 sec) x 40, with a melting temperature ramp following amplification. A robotic system was used to load a 384 well plate which was then run on the ABI 7900 quantitative real time PCR machine. All samples were tested in triplicate. The expression levels were calculated relative to the housekeeping gene *Pgk* or *18s* ribosomal RNA (Qm18s primers). The expression results are the average of 2 to 5 separate PCR amplifications and the average of the results using the two different housekeeping genes. When gene expression result relative to *Pgk* and the gene expression relative to *18s* ribosomal RNA (*18s* rRNA) for the individual samples differed by more than two fold, the samples were eliminated from the average due to the ambiguity of the cDNA quantity. Change in expression after irradiation was determined by comparing the relative gene expression of the irradiated right leg with the non-irradiated left leg of the individual mice for genes associated with the development of fibrosis in skin and muscle. This included *Acta2*, the gene coding for alpha smooth muscle activin (SMA), *Tgfb1*, a known fibrogenic cytokine, *Col1a1*, gene coding for a collagen 1, and the *Inhba* gene that encodes for the inhibin βA subunit, a component of activin A.

### Histology

Tissues were fixed in 10% formalin, washed in ethanol and embedded in paraffin. Sections were stained with haematoxylin and eosin. Epidermal thickness was measured with at least 20 measurements spanning approximately 3 mm length of skin using Aperio ImageScope software (Aperio digital pathology, Leia Biosystems, Nussloch, Germany). The area of the nucleus of the epidermal cells was calculated by measuring the width and length and determined using the formula for the area of an ellipse (π x width x length / 4). The width and length were measured using the Aperio ImageScope and 100 nuclei were measured in each section (7 to 9 legs were used for each group).

### Statistics and development of fibrosis prediction assay

A one-tailed, Mann-Whitney U test was conducted to determine if there was either an increase or decrease between specified groups for gene expression and comparisons between control and follistatin treated mice. The one-tailed paired Wilcoxon signed-rank test was used to test for either an increase (for epidermis thickness and nuclei size) or decrease (leg extension) after irradiation when comparing the left to the right legs. P values less than 0.05 were designated to be statistically significant.

## Results

**Fig 1:**
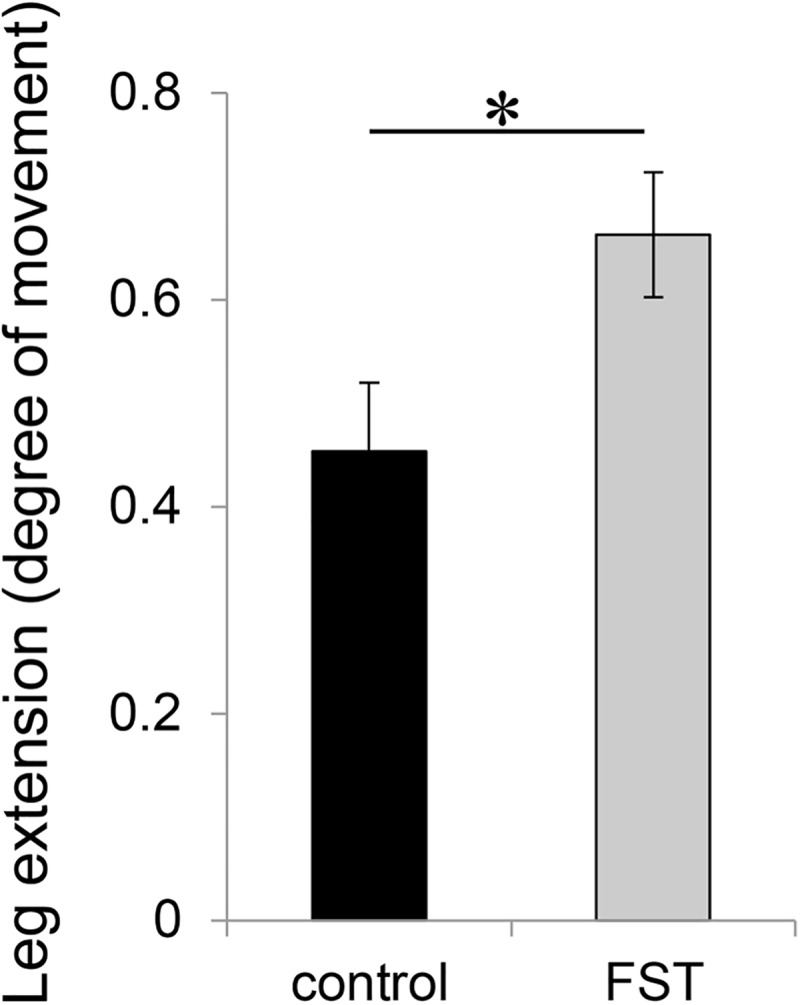
Follistatin treatment of irradiated mouse legs improve extension in a fibrosis model at 6 months post-IR. Mice were irradiated with 35 Gy and leg extension was tested to measure leg flexibility at 6 months post-IR. The leg extension (change in leg extension angle following radiation) was calculated by dividing the extension angle of the irradiated right leg with the extension angle of the non-irradiated left leg. The legs from the follistatin treated mice (FST; grey bar) were compared to the legs from the PBS (control; black bar) treated mice. *p = 0.03; n = 8 and 9 for controls and FST, respectively; SEM.

Serum follistatin levels rose significantly (p = 0.003, n = 9) following the subcutaneous injection of follistatin over the course of 6 months (S1 Fig). The irradiated legs, when compared to the non-irradiated legs in the mice injected with PBS showed a statistically significant decrease in leg extension as expected for this model (p<0.01, n = 8; Fig 1) [29]. The mice treated with follistatin also had a statically significant decrease in irradiated leg extension (p<0.01, n = 9; Fig 1) but demonstrated a significant increase in the amount of extension compared to the PBS injected irradiated legs (p = 0.03, n = 8 for controls and n = 9 for follistatin treated; Fig 1).

**Fig 2:**
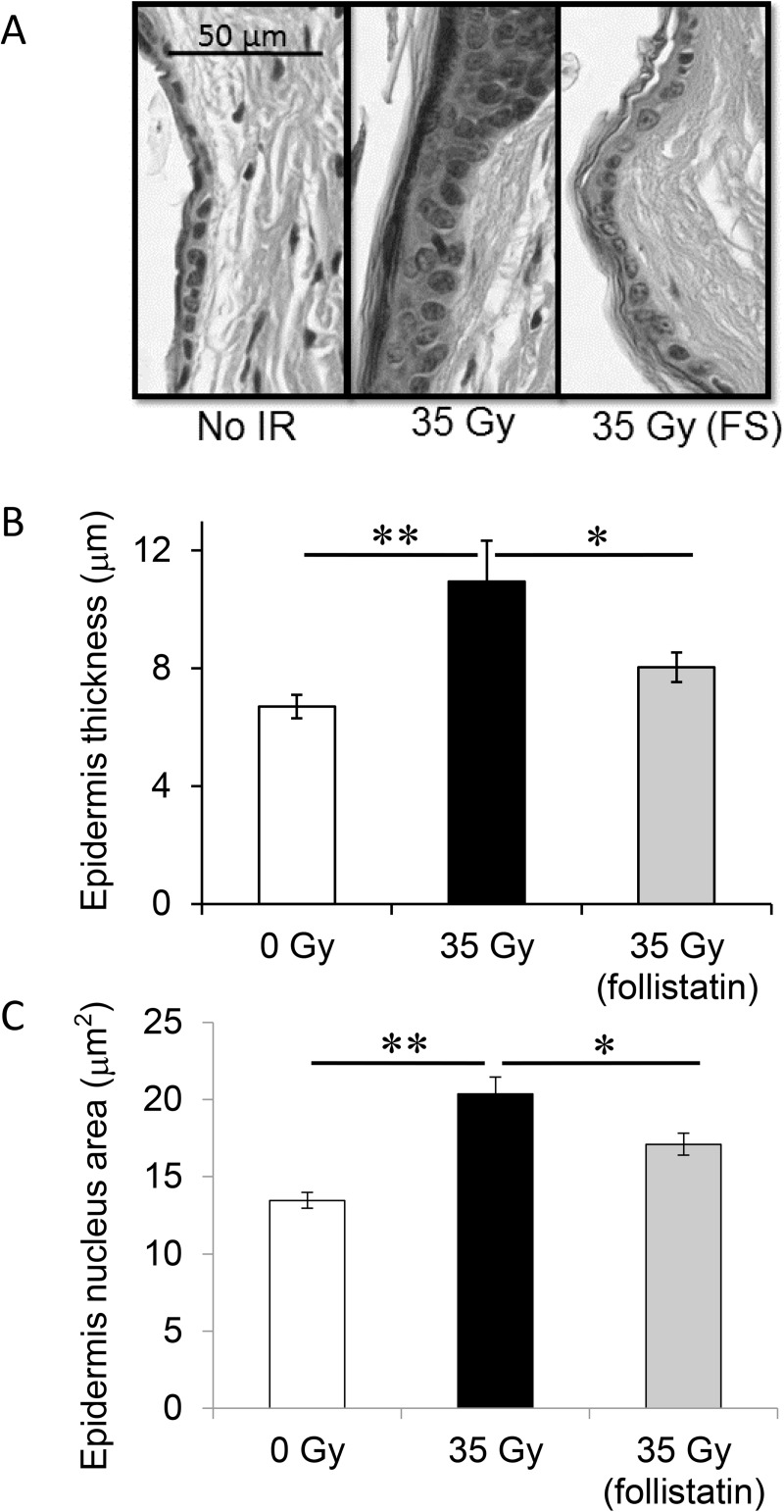
Epidermal thickness and nuclei size are increased 6 months after irradiation, are decreased with follistatin, and can be used as a marker for fibrosis. Mouse legs were irradiated with 35 Gy and skin tissue was harvested 6 months after irradiation. Sections were stained with haemotoxylin and eosin and epidermis thickness was measured (upper panel). No IR tissue section and 0 Gy results are from the left, non-irradiated legs of PBS injected mice. Measurements of epidermis thickness are plotted (middle panel) and increase after irradiation in PBS injected mice (p<0.01). The irradiated epidermis of mice treated with follistatin is significantly thinner (35Gy (follistatin), p = 0.03). The lower panel shows the measurement of the nucleus area in the epidermis. The size of the nuclei increased after irradiation (p = 0.0005), however, the nucleus size after irradiation is significantly less in the follistatin treated mice (p = 0.03). *p<0.05; **p<0.01; n = 8 to 9n. Error bars represent SEM.

Further, the measurements of the epidermis thickness from the irradiated region of the leg showed that there was a significant increase in the irradiated leg compared to the non-irradiated leg (p<0.01, n = 8; Fig 2). In the follistatin treated mice, there was also a significant increase in the thickness of the epidermis in the irradiated leg (p<0.01, n = 9; Fig 2). However, there was a significant decrease in the amount of epidermal thickening resulting from the irradiation in the follistatin treated mice (p = 0.03, n = 8; Fig 2). We also observed a significant increase in the size of the nuclei in the epidermal cells after irradiation in PSB injected mice (p<0.01, n = 8; Fig 2). There was no significant difference in the size of the nuclei in the epidermis cells after irradiation in the follistatin treated mice; and the size of the nuclei in the irradiated legs was significantly less in the follistatin treated mice compared to the PBS injected mice (p = 0.03, n = 8; Fig 2).

**Fig 3:**
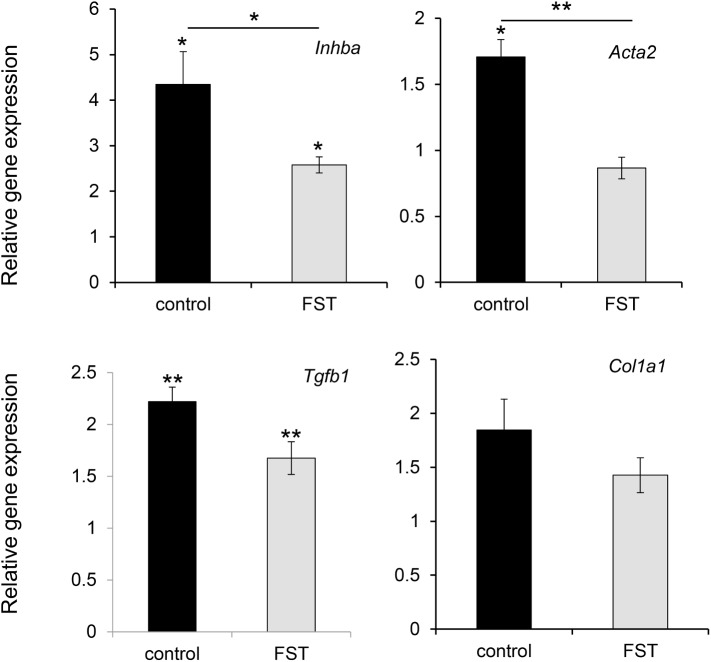
The difference in increase in expression in muscle 6 months after 35 Gy of irradiation for *Inhba Acta2*, *Tgfb1*, and *Col1a1* between mice injected with PBS (control) and follistatin (FST). The expression increase was determined by comparing the expression in the muscle of irradiated with non-irradiated leg for each individual mouse. The statistical significant difference of expression is indicated directly above the bars and the statistical significance for the difference between the irradiated groups is shown above the line between the groups. *p<0.05; **p<0.01, no asterisk, p>0.05; n = 5 to 8; error bars represents SEM.

*Inhba* codes for the inhibin subunit b, a component of activin A which is directly inhibited by follistatin and is also involved with fibrosis. In muscle, *Inhba* was significantly increased approximately 4.3-fold in the irradiated leg of the control (PBS only) mice (p = 0.0009, n = 6; Fig 3) and 2.6 fold in the follistatin treated mice (p = 0.005, n = 6; Fig 3). In follistatin treated mice, the relative increase in *Inhba* expression after irradiation was less than for control mice (p = 0.047, n = 6; Fig 3). A marker of activated fibroblasts, *Acta2*, showed an increase in irradiated muscle (p = 0.008, n = 6: Fig 3), but no significant increase was seen in irradiated legs of follistatin treated mice. The relative increase in *Acta2* gene expression after irradiation in follistatin treated mice was significantly less than for the control mice (p = 0.004, n = 6; Fig 3). There was a small increase in *Acta2* expression in the non-irradiated muscle of the follistatin treated mice compared to control mice (less than 1.4 fold, p = 0.02; data not shown). In the irradiated leg, the expression level of *Tgfb1* significantly increases in the muscle of the control mice (p = 0.0004, n = 8; Fig 3) as well as in the follistatin treated mice (p = 0.004, n = 8; Fig 3). No statistically significant increase in *Col1a1* expression was observed 6 months after irradiation in the muscle (Fig 3).

**Fig 4:**
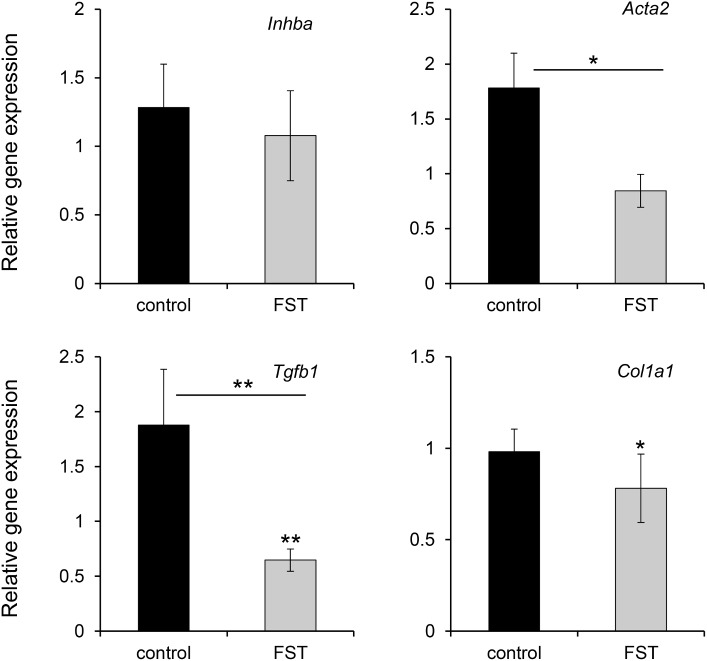
The difference in increase in expression in skin 6 months after 35 Gy for *Inhba*, *Acta2*, *Tgfb1*, and *Col1a1* between mice injected with PBS (control) and follistatin (FST). The expression increase was determined by comparing the expression in the skin of irradiated with the non-irradiated leg for each individual mouse. The statistical significant difference in expression is indicated directly above the bars and the statistical significance for the difference between the irradiated groups is shown above the line between the groups. *p<0.05; **p<0.01 no asterisk, p>0.05; n = 8 to 9; error bars represents SEM.

The gene expression in the skin showed some similar trends to the muscle but there were also some differences. Unlike the muscle, there was no significant increase observed in *Inhba* expression in the skin after irradiation (Fig 4). Although the increase in *Acta2* expression in the skin after irradiation was not significant, the relative increase of *Acta2* expression in the skin after irradiation in follistatin treated mice was significantly less than for control mice (p = 0.015, n = 8; Fig 4). There was no significance for an increase in *Tgfb1* in the skin after irradiation in the control mice (Fig 4). However, there was a significant decrease in the *Tgfb1* expression in the skin after irradiation of follistatin treated mice (p = 0.009; n = 9; Fig 4). This was partially due to a small increase in *Tgfb1* expression in skin of non-irradiated leg of follistatin treated mice (less than 1.6 fold; p = 0.03) (data not shown). The relative levels of *Tgfb1* expression in the skin after irradiation of the follistatin treated mice was significantly less than for control mice (p = 0.004, n = 9; Fig 4). Similar to the muscle, no increase in *Col1a1* expression was observed 6 months after irradiation in the skin (Fig 4). However, a small significant decrease was observed in the skin after irradiation in the follistatin treated mice (p = 0.02, n = 9; Fig 4).

## Discussion

These physical, histological and molecular investigations have shown that follistatin, a potent inhibitor of the actions of activin, can ameliorate the fibrotic endpoints in a radiation-induced mouse model for fibrosis. These beneficial effects of follistatin were demonstrated by the measurements of leg extension and modulation of skin thickness and nuclear features of the epidermal cells caused by irradiation. Further, in the follistatin treated mice, the biochemical markers of fibrosis such as the expression of extracellular matrix genes that code for SMA (*Acta2*) is correspondingly lower consistent with the physical measurements of contraction. The increased thickness of the skin and extracellular matrix contributes to the reduced movement in the leg and reduction of these factors due to follistatin treatment would improve the flexibility. In addition, the molecular analysis indicates that the expression of genes of the major fibrosis-associated factors such as activin A (*Inhba*) is upregulated in the muscle of irradiated fibrotic tissue and that follistatin attenuates this response. This also indicates that there is less inflammation in the follistatin treated mice 6 months after irradiation.

*Acta2* gene expression increased to a similar degree in the skin and muscle of irradiated legs of control mice indicating inflammation. However, the increase in *Inhba* expression levels in the irradiated leg increases approximately 4 fold in the muscle, but did not increase in the skin indicating differences between the fibrotic inflammation responses in the different tissue types. The follistatin treatment still significantly decreases the *Tgfb1* expression in the irradiated skin indicating that activin A is involved in the increased *Tgfb1* expression in the skin. The quality of the skin RNA is inferior due to the presence of degraded RNA from the dead skin layer. Differences in the percentage of degraded RNA may vary from sample to sample and may contribute to the variation of gene expression observed between individuals within each group compromising the sensitivity of this assay. Differences in ratio of cell types within the different skin samples may also contribute to the variation.

Several methods have been used to induce fibrosis in animal models; these include bleomycin treatment [24], fibrogenic proteins [30], and radiation [29,31,32]. In this study, we have utilized the radiation-induced fibrosis model since it is the most clinically relevant for radiotherapy outcomes. The fibrosis induced by ionising radiation in our study emulates the long-term progression of fibrosis that is observed following radiotherapy in humans. The radiation-induced fibrosis mouse model is also well-characterized, dose dependent and thus provides an excellent model to investigate the role of the activins and follistatin in fibrogenesis [33,34]. Therefore, the promising data generated through the present series of studies contribute to the effort to develop an effective treatment for radiation-induced fibrosis.

TGFβ has been identified in many studies to be a key driver of fibrosis, but it has a complex modulatory role and can play both pro- and anti-fibrotic roles [8,35,36]. Although TGFβ1 is associated with fibrosis and its levels are often associated with the severity of the fibrosis, it modulates immune responses and tissue repair [8]. It has been found to enhance wound healing [37] but prolonged exposure can lead to fibrosis [30]. Furthermore, the study which demonstrated that follistatin could attenuate the TGFβ induced fibrosis strongly suggests that the actions of TGFβ are expressed by the induction of activin A, since follistatin cannot physically bind TGFβ [10,11,38].

Currently there is no effective Food and Drug Administration (FDA)-approved treatment for radiation-induced fibrosis. However, some treatments for pulmonary fibrosis show promising results. Halofuginone inhibits various members of the TGFβ signalling pathway, the synthesis of type I collagen, and phosphorylation of SMAD3. Halofuginone caused a significant decrease in fibrosis in radiation-treated mice [31], but side effects such as bleeding, nausea, vomiting and fatigue have been reported with high inter-patient variability [39]. Pirfenidone, and other broad spectrum compounds that modulate the TGFβ superfamily show promise for treating fibrosis, and pirfenidone is an approved treatment in several countries for mild to moderate idiopathic pulmonary fibrosis. However, the long term efficacy and safety of pirfenidone has recently been questioned with other side effects such as nausea, hepatic dysfunction, weight loss, dizziness and fatigue [40]. Recently, a TGFβ receptor I kinase inhibitor has been shown to have beneficial effects on pulmonary fibrosis [32]. While modulating activin bioactivity reduces fibrosis in several models, its efficacy has not been tested in a radiation-induced fibrotic model.

The clear demonstration of the effectiveness of follistatin in decreasing the physical limitations in the irradiated mice raises an exciting prospect that treatment of patients undergoing radiation with follistatin could prevent fibrosis, and also facilitate the use of radiation dose escalation. The successful translation of these results from a murine model to humans has the potential to transform the management of radiation–induced fibrosis. The results of these studies could also translate to other organs where radiation-induced fibrosis occurs, and provide a means to improve radiotherapy outcomes. The translation of these preclinical results to the clinic is feasible since the present study and toxicity testing have shown no adverse effects of follistatin treatment. We acknowledge that further optimization in timing and dose of follistatin administration could improve the present results beyond the findings reported here.

We conclude that follistatin may be a potential novel therapeutic agent for the prevention of severe fibrosis after radiotherapy and should be further studied.

## Supporting information

S1 Fig Levels of follistatin (ng / ml) in mouse serum 6 months after 35 Gy were statistically significantly higher in those mouse subcutaneously injected with follistatin (p<0.001). Levels of activin A (pg / ml) in the serum was not significantly different between the control and follistatin treated mice (p = 0.99). n = 9; error bars represent SEM.
(PDF)
