# Supplementary figures and images for: Follistatin attenuates radiation-induced fibrosis in a murine model
Source: PLoS One. 2017 Mar 16;12(3):e0173788. doi: 10.1371/journal.pone.0173788 (PMC5354399; doi:10.1371/journal.pone.0173788)

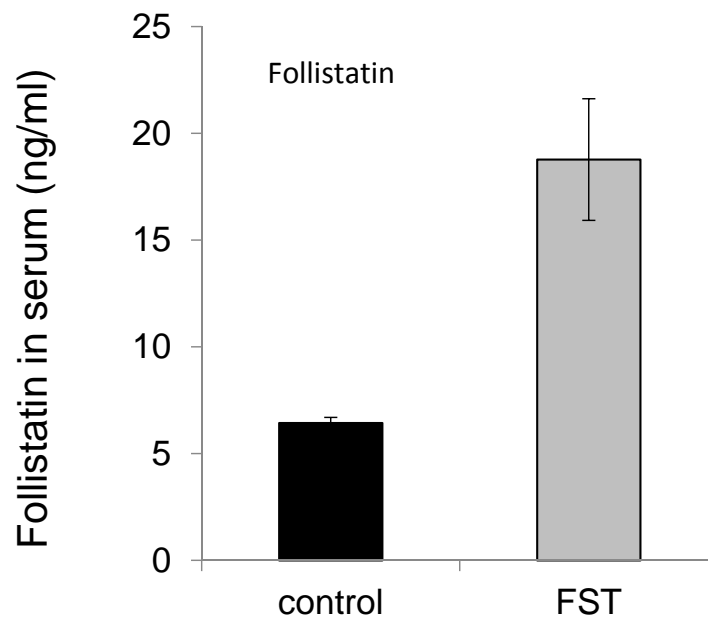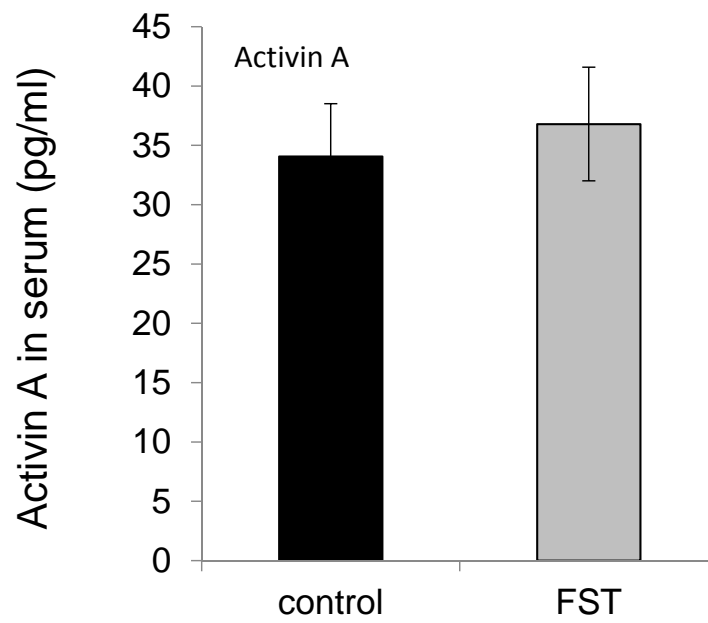

Figure S1

Supplement: S1 Fig — Levels of activin A (pg / ml) in the serum was not significantly different between the control and follistatin treated mice (p = 0.99). n = 9; error bars represent SEM. (PDF) [file pone.0173788.s001.pdf]
